# Efficacy and Safety of Selective Internal Radiation Therapy (SIRT) for Liver Metastases in Breast Cancer: An Umbrella Review

**DOI:** 10.3390/cancers18050756

**Published:** 2026-02-26

**Authors:** Marco Cuzzocrea, Stefano Cappio, Marzia Conti Beltraminelli, Lorenzo Rossi, Chiara Martinello, Giorgio Treglia, Federico Pedersoli, Gaetano Paone

**Affiliations:** 1 Department of Nuclear Medicine, Istituto di Diagnostica Integrata della Svizzera Italiana (IDISI), Ente Ospedaliero Cantonale (EOC), Via Athos Gallino 12, 6500 Bellinzona and Via Tesserete 46, 6900 Lugano, Switzerland; marco.cuzzocrea@eoc.ch (M.C.); chiara.martinello@eoc.ch (C.M.); gaetano.paone@eoc.ch (G.P.); 2 Department of Radiology, Istituto di Diagnostica Integrata della Svizzera Italiana (IDISI), Ente Ospedaliero Cantonale (EOC), 6900 Lugano, Switzerland; stefano.cappio@eoc.ch; 3 Centro di Senologia della Svizzera Italiana (CSSI), 6962 Lugano, Switzerland; marzia.contibeltraminelli@eoc.ch (M.C.B.); lorenzo.rossi@eoc.ch (L.R.); 4 Istituto Oncologico della Svizzera Italiana (IOSI), Ente Ospedaliero Cantonale (EOC), 6962 Lugano, Switzerland; 5 Faculty of Biomedical Sciences, Università della Svizzera Italiana (USI), Via Buffi 13, 6900 Lugano, Switzerland; 6 Faculty of Biology and Medicine, University of Lausanne, Rue du Bugnon 21, 1011 Lausanne, Switzerland; 7 Department of Radiology, ASST Spedali Civili di Brescia, Piazzale Spedali Civili 1, 25123 Brescia, Italy; federico.pedersoli@asst-spedalicivili.it

**Keywords:** breast cancer, SIRT, TARE, radioembolisation, metastases

## Abstract

**Background/Objectives**: Liver metastases in breast cancer patients are associated with poor prognosis and limited therapeutic options. Selective Internal Radiation Therapy (SIRT), also known as transarterial radioembolization (TARE), has emerged as a loco-regional treatment modality, particularly in cases refractory to systemic therapies. **Objective**: To systematically review and synthesize evidence from existing systematic reviews and meta-analyses on the efficacy and safety of SIRT in breast cancer patients with liver metastases. **Methods**: A comprehensive literature search was conducted in PubMed using predefined keywords related to SIRT and breast cancer, restricted to systematic reviews and meta-analyses. Inclusion criteria were reviews evaluating SIRT in breast cancer patients with hepatic metastases, reporting on efficacy (e.g., objective response rate, disease control rate, overall survival) and/or safety outcomes. The quality of included reviews was assessed using AMSTAR 2. **Results**: Seven systematic reviews and meta-analyses were included. Reported objective response rates (ORRs) ranged from 36% to 61%, and disease control rates (DCRs) from 78 to 96%. Toxicity profiles were generally favorable, with limited grade 3–4 adverse events. Some heterogeneity was noted in patient selection, types of microspheres used (glass vs. resin), and outcome definitions. **Conclusions**: SIRT appears to be a promising option for breast cancer patients with liver metastases, particularly in the setting of treatment resistance. However, heterogeneity among available studies and the lack of prospective randomized trials limit definitive conclusions. Further high-quality studies are warranted.

## Simple Summary

Hepatic metastases are a frequent manifestation in advanced breast cancer and are associated with limited therapeutic options and poorer prognosis. Selective Internal Radiation Therapy (SIRT) is a locoregional treatment that delivers radiation directly to liver tumors through the bloodstream. This umbrella review summarizes the results of several previous studies that analyzed how effective and safe SIRT is for patients with breast cancer that has spread to the liver. The findings suggest that SIRT can help control the disease in many cases and has manageable side effects. However, more high-quality research is needed to fully understand how and when to use this treatment.

## 1. Introduction

Breast cancer is the most commonly diagnosed cancer among women worldwide and a leading cause of cancer-related death [1]. Although advances in systemic therapy have improved outcomes, metastatic disease—particularly liver metastases—remains a major clinical challenge. Based on population data from the Surveillance, Epidemiology, and End Results (SEER) database, the incidence of liver as the first metastatic site is 25.6% among breast cancer patients. Similarly, an institutional cohort from the Fudan University Shanghai Cancer Center (FUSCC) reported an incidence of first liver metastases of 35.6%. Median overall survival for patients with breast cancer liver metastases (BCLMs) remains poor, typically ranging from 20.0 months (SEER) to 27.3 months (FUSCC), with significant variation based on molecular subtype and available therapies (e.g., HER2-targeted therapy) [2].

Molecular subtypes notably influence metastatic behavior and survival outcomes. Specifically, HER2-positive and triple-negative breast cancers exhibit a higher propensity for liver dissemination compared to luminal subtypes [3,4].

Therapeutic strategies for managing BCLM include systemic treatments (chemotherapy, endocrine therapy, and HER2-targeted agents), surgical resection, local ablative techniques (radiofrequency ablation, microwave ablation), hepatic arterial infusion (HAI), transarterial chemoembolization (TACE), and Selective Internal Radiation Therapy (SIRT). The choice of treatment often depends on tumor biology, disease burden, patient characteristics, and the availability of treatments [2,3,4]. In this context, SIRT has emerged as a potential loco-regional treatment option. SIRT involves the intra-arterial administration of microspheres loaded with yttrium-90, delivering high-dose radiation directly to liver tumors while sparing normal parenchyma. The technique is widely used in primary and secondary liver malignancies [5], and its application in breast cancer liver metastases (BCLMs) has gained increasing attention [6], although it is not yet well recognized as a therapeutic option, perhaps in relation to the radiosensitivity of metastases to conventional radiotherapy [7].

We conducted an umbrella review—an overview of systematic reviews—to assess the efficacy and safety of SIRT in breast cancer. This review provides a comprehensive evaluation of its utility in BCLM and its broader clinical implications, drawing on data of the highest level of evidence from systematic reviews and meta-analyses.

## 2. Materials and Methods

This umbrella review was performed according to a predefined protocol [8] and following PRISMA guidelines to ensure the reproducibility of our search and selection process. The protocol was not registered on PROSPERO or public databases.

Based on a predefined research question (“What is the efficacy and safety of Selective Internal Radiation Therapy in breast cancer liver metastases?”), a search string was created using a combination of “free-text” keywords and Boolean operators. The complete search string used for the literature search was: (A) “breast” AND (B) radioembolization OR Selective Internal Radiation Therapy OR SIRT OR transarterial radioembolization OR TARE AND (C) “review” OR “systematic review” OR “meta-analysis” OR “evidence-based”. An electronic web-based comprehensive search of the medical literature was carried out using the above-listed search string on two different bibliographic databases (PubMed/MEDLINE and Cochrane Library). The last update of the literature search was on 3 July 2025. No language restrictions or limitations concerning the year of publication were applied. References of the retrieved records were screened, searching for additional systematic reviews or meta-analyses on the selected topic. Regarding the inclusion criteria, only systematic reviews with or without meta-analyses investigating the efficacy and safety of SIRT in BCLM were eligible for inclusion in our umbrella review. Two review authors (MC and GT) independently performed the literature search and the selection of eligible records by applying the inclusion criteria mentioned above, and the data extraction and the quality assessment of eligible systematic reviews according to the AMSTAR-2 tool [9]. For each selected systematic review (with or without meta-analysis), information was collected, including authors, year of publication, number of original articles included, patients included, and main findings (with a special focus on diagnostic performance data).

## 3. Results

### 3.1. Literature Search Results

The comprehensive web-based literature search using PubMed/Medline and Cochrane Library databases retrieved 54 records. Following the screening process according to the predefined inclusion and exclusion criteria, 7 systematic reviews and/or meta-analyses were selected for inclusion in this review [10,11,12,13,14,15,16]. The quality assessment of included evidence-based articles according to AMSTAR-2 is illustrated in Table 1. Four reviews were rated as low quality [10,11,12,13] and three as moderate quality [14,15,16]. Limitations included a lack of protocol registration, insufficient reporting of risk of bias in primary studies, and heterogeneity in outcome reporting. Given that four of seven included evidence syntheses were rated as low quality by AMSTAR-2, the quantitative ranges reported in the tables should be interpreted cautiously and primarily as hypothesis-generating.

The main characteristics of selected systematic reviews or/and meta-analyses are presented in Table 2 and Table 3 and summarized below.

### 3.2. Efficacy Outcomes

Across the included evidence syntheses [10,11,12,13,14,15,16], SIRT demonstrates a consistent “class effect” on tumor control. Pooled analyses place objective response rates (ORRs) broadly in the 36–51% range and disease control rates (DCRs) around 85–88%, with higher estimates when using metabolic response criteria. Specifically, Liu et al. reported pooled ORR/DCR by response framework—RECIST ORR 36% (95% CI 26–47) with DCR 85% (76–93), mRECIST ORR 49% (34–65) with DCR 73% (59–85), and PERCIST ORR 47% (17–78) with DCR 97% (91–100) [15]. In a complementary meta-analysis focused on response, Quartuccio et al. found a pooled ORR of 50.7% (40.0–61.4) and DCR of 88.4% (81.9–93.6) [16]. Earlier systematic work by Smits et al. reported ORR 39–61% and DCR 78–96% in a historical series [10], consistent with these ranges, while Feretis et al. estimated a DCR of 81% across 12 non-randomized studies [11].

Survival outcomes are likewise convergent but heterogeneous across settings. Liu et al. estimated a post-embolization median survival of 9.8 months (95% CI 9.0–11.6) [15], whereas Rivera et al., summarizing 10 SIRT series, reported a median overall survival of 11.5 months (range 5–26) [12]. Aarts et al.’s comparative meta-analysis (SIRT/hepatic arterial infusion, HAI) yielded a pooled SIRT ORR of 49% (32–67) with a median survival of 9.2 months (range 6.1–35.4) but substantial heterogeneity (I^2^ ≈ 65%), underscoring variability in patient selection, techniques, and endpoints [14]. Narrative syntheses, including Guglielmo et al., reflect similarly broad survival ranges (≈6.1–35.4 months) and emphasize between-study diversity [13].

Subgroup analyses consistently identified disease extent and hepatic tumor burden as key modifiers (as shown below). The highest survival rate reported across series was observed in cohorts enriched for liver-only or liver-dominant disease and lower hepatic involvement, while outcomes are poorer in more disseminated or high-burden settings.

### 3.3. Safety Outcomes

Across studies, SIRT was generally well tolerated. Liu et al. conducted a pooled safety analysis of SIRT in breast cancer patients with liver metastases, including 480 patients across 10 studies [15]. Post-embolization complications were relatively infrequent and mostly mild. Cholecystitis occurred in 1.5% of patients, all grade ≤ 2. Gastrointestinal ulcers were observed in 3.0% of patients, with 2.1% being grade ≥ 3. Grade 3 pancreatitis was reported in 0.4% of cases. Regarding biochemical toxicities, grade ≥ 3 elevations in aminotransferases occurred in 18.1% of patients, while hyperbilirubinemia was seen in 6.3%. Other grade ≥ 3 toxicities included elevated alkaline phosphatase (4.4%) and leukocytosis (3.3%), and there were no reported cases of grade ≥ 3 anemia or thrombocytopenia. It is important to note that the number of patients assessed for each toxicity varied significantly across included studies. Higher toxicity was associated with larger tumor burden and the presence of extrahepatic disease. Aarts et al. reported adverse events in 11 of 26 studies included in their analysis, but did not stratify them by treatment type due to the different criteria methods used as endpoints, as some studies incorporated laboratory results and others only clinical ones [14]. Feretis and Rivera provided only qualitative safety descriptions without pooled estimates [11,12].

Smits et al. highlighted common post-procedure symptoms (e.g., abdominal pain, nausea, gastric ulcers) and reported rare but severe events, including three treatment-related deaths attributed to radioembolization-induced liver disease (RILD) within 3 months after SIRT; histopathologic confirmation was not reported [10]. Guglielmo et al. [13] indicated mostly mild to moderate adverse events, with severe toxicity being rare and no grade 4 hepatic toxicity (Table 3).

### 3.4. Subgroup Analyses

Several included meta-analyses provided subgroup analyses that offer important insights into factors influencing treatment outcomes with SIRT in patients with liver metastatic breast cancer.

#### 3.4.1. Comparison with Other Local Treatments

Aarts et al. conducted a subgroup analysis comparing three different intra-arterial liver-directed therapies: SIRT, TACE, and HAI. The reported ORRs were 49% for SIRT, 34% for TACE, and 19% for HAI. Pooled median OS was 9.2 months for SIRT, 17.8 months for TACE, and 7.9 months for HAI. Despite heterogeneity in patient selection and study designs, SIRT and TACE showed comparable disease control, while HAI appeared less effective in terms of response rate. No comparison could be made for OS due to the lack of survival rates at specific time points (1- and 2-year OS) and the high degree of heterogeneity [14].

Rivera et al. also evaluated the efficacy of multiple liver-directed therapies, including hepatic resection, radiofrequency ablation (RFA), TACE, HAI, and SIRT. While limited by the lack of pooled quantitative synthesis, the authors suggested that hepatic resection and RFA were associated with the most favorable outcomes in selected patients, with median OS reaching up to 45 months in resected patients and up to 38 months for those treated with RFA [12].

#### 3.4.2. Type of Microspheres (Resin vs. Glass)

Quartuccio et al. performed a subgroup analysis comparing resin and glass microspheres. Patients treated with resin microspheres showed significantly higher response rates: ORR was 60.4% (95% CI: 50.6–69.5) and DCR 92.7% (95% CI: 86.5–96.3), compared to ORR 32.4% (95% CI: 23.4–42.9) and DCR 82.7% (95% CI: 72.9–89.5) in the glass microsphere group [16].

#### 3.4.3. Response Criteria (RECIST, mRECIST, PERCIST)

Liu et al. assessed outcomes based on different response criteria. Using RECIST, the pooled ORR was 36%, and DCR 85%; using mRECIST, ORR was 49% and DCR 73%; and with PERCIST, ORR was 47%, and DCR reached 97%. These differences highlight how response assessment methodology can substantially affect reported efficacy [15].

#### 3.4.4. Hepatic Tumor Burden (<25% vs. >25%)

Patients with a hepatic tumor burden < 25% had a median OS of 10.5 months (95% CI: 8.9–13.4), whereas those with >25% involvement had significantly shorter OS of 6.8 months (95% CI: 5.5–8.9), indicating tumor load as a key prognostic factor [15].

#### 3.4.5. Presence of Extrahepatic Metastases

Liu et al. also stratified survival by metastatic spread. Patients without extrahepatic disease achieved a median OS of 15.0 months (95% CI: 12.5–17.6), compared to 5.3 months (95% CI: 4.1–6.5) in patients with extrahepatic metastases. These data confirm that isolated liver involvement is associated with better prognosis and may represent the most favorable setting for SIRT [15].

#### 3.4.6. Correlation Between BC Hormone Receptor Status and Response

In their analysis, Quartuccio et al. found no significant correlation between breast cancer hormone receptor status and either ORR or DCR (*p* > 0.05) [16].

## 4. Discussion

This umbrella review synthesized and critically appraised seven evidence-based studies evaluating the efficacy and safety of Selective Internal Radiation Therapy (SIRT) in patients with breast cancer and liver metastases [10,11,12,13,14,15,16]. While the overall findings suggest a promising therapeutic role for SIRT in this setting, several methodological and clinical issues limit the strength and generalizability of the conclusions.

Across studies, disease control rates (DCRs) ranged from 78% to 96%, while objective response rates (ORRs) varied from 36% to 61%. This heterogeneity in reported efficacy is partly attributable to differences in microsphere type, imaging response criteria, patient characteristics, and outcome definitions. Resin microspheres were associated with higher ORR and DCR compared to glass microspheres, as demonstrated in the meta-analysis by Quartuccio et al. [16]. The apparent differences in response outcomes between resin and glass microspheres (higher ORR/DCR in resin-treated cohorts) should be interpreted with restraint. These comparisons are accompanied by substantial heterogeneity across studies (I^2^ ≥ 72% for all outcomes) and, critically, occur in the absence of individualized absorbed-dose information. Without patient-specific dosimetry and standardized treatment planning, observed differences may reflect confounding by patient selection, baseline tumor burden, vascular anatomy, activity prescription strategies, and imaging response assessment rather than microsphere type. Accordingly, microsphere comparative findings should be considered hypothesis-generating rather than definitive and in need of confirmation in dosimetry-driven prospective studies.

Similarly, the use of functional imaging criteria (PERCIST) yielded notably higher DCR compared to traditional anatomic criteria (RECIST or mRECIST), underscoring the influence of response assessment methods on outcome interpretation [15]. Metabolic criteria (PERCIST) capture changes in tumor metabolism that may precede size reduction and can therefore yield higher DCR compared with anatomic criteria (RECIST/mRECIST). However, higher metabolic response rates do not necessarily translate into improved survival or durable clinical benefit, particularly in heavily pre-treated populations. In Liu et al., DCR increased from 73% with mRECIST to 97% with PERCIST, while ORR remained broadly comparable across criteria. This discrepancy is clinically plausible because PERCIST captures metabolic changes that may precede, or occur without, measurable tumor shrinkage on anatomic imaging, particularly in the setting of therapy-induced necrosis, edema, or mixed morphologic changes after intra-arterial therapy. In heavily pre-treated populations, metabolic response may reflect early biological effect while subsequent systemic progression (including extrahepatic progression) remains a competing driver of outcome. Therefore, response criteria should be explicitly reported and interpreted as method-dependent endpoints rather than interchangeable measures of efficacy.

Prognostically, the most reproducible modifiers are disease extent and hepatic tumor burden. Nonetheless, interpretation of survival outcomes requires particular caution. Across included reviews, median OS values are broadly in the 9–12 month range in many SIRT series, but reported ranges extend to about 35 months, reflecting substantial heterogeneity and likely selection or lead-time bias. Importantly, these figures are strictly descriptive in the absence of randomized comparisons, appropriate control cohorts, or consistently adjusted analyses and cannot be interpreted as evidence of survival benefit attributable to SIRT. Similarly, it is not possible to compare them with the results of alternative local or systemic therapies across different clinical contexts. Granular subgroup findings reinforce that prognosis is driven primarily by disease extent and hepatic tumor burden. In Liu et al. [15], median OS was 15.0 months in patients without extrahepatic metastases versus 5.3 months in those with extrahepatic disease and 10.5 months for tumor burden < 25% vs. 6.8 months for >25%. These gradients strongly suggest that the most favorable survivals occur in cohorts enriched for liver-only or liver-dominant disease and limited hepatic involvement, whereas outcomes are worse in disseminated or high-burden settings.” These findings highlight the importance of careful patient selection—biology, burden, and distribution—when considering SIRT. As highlighted by our analysis of evidence-based studies, breast cancer patients who could be excellent candidates for SIRT treatment are those with oligometastatic disease with a predominantly hepatic disease burden. It should be noted that, considering the heterogeneity of treatment response and survival data, the decision to perform treatment with SIRT should be based on a multidisciplinary decision on its primary purpose, which is local consolidation of ongoing systemic treatment rather than replacement or palliative care in selected cases.

Safety data were inconsistently collected and often qualitatively described, limiting the ability to draw firm conclusions on toxicity. Only one review [15] systematically examined adverse events, demonstrating that the incidence rate of major gastrointestinal complications, such as ulcer, cholecystitis, and pancreatitis, was less than 3%, with no procedure-related deaths. In contrast, several other syntheses provided qualitative descriptions without pooled estimates, limiting cross-study comparability. A balanced interpretation must also acknowledge the rare but serious adverse event reported by Smits et al., namely three treatment-related deaths attributed to RILD within 3 months after SIRT, although it was not possible to assess whether this diagnosis was confirmed histopathologically [10]. The risk of radioembolization-induced liver disease may occur particularly in patients who have received multiple prior systemic therapy lines, as cumulative chemotherapy exposure can increase hepatic radiosensitivity and reduce functional reserve. Furthermore, unlike HCC, where one of the most important risk factors is cirrhosis, RILD in breast cancer patients is often caused by chemotherapy-associated steatohepatitis (CASH). Careful patient selection and pre-treatment evaluation of liver function are therefore crucial to minimize this risk [17]. In this connection, clinicians should monitor for hepatobiliary complications, particularly in patients with preexisting liver dysfunction.

In comparative studies of intra-arterial options, SIRT and TACE achieve higher response rates than HAI, while survival comparisons are hampered by heterogeneity and selection differences [14]. Broader systematic reviews indicate that hepatic resection—and, in carefully selected oligometastatic cases, RFA—can achieve the longest survivals, whereas SIRT/TACE are most often used in unresectable, liver-dominant, or chemo-refractory disease settings [12]. These survival differences largely reflect clinical selection rather than the intrinsic superiority of the modality. Patients offered hepatic resection or, in strictly oligometastatic settings, RFA typically have lower hepatic tumor burden and smaller/limited lesions, all factors associated with longer survival; by contrast, SIRT/TACE cohorts are commonly unresectable, liver-dominant, or chemo-refractory, which decreases median OS. Against non–intra-arterial treatments, external-beam radiotherapy is typically positioned as adjunctive rather than definitive for this indication, while systemic therapies remain the backbone of care [18]; within this landscape, SIRT provides liver-specific disease control that can complement systemic treatment in appropriately selected patients [12,14] and postpone the subsequent therapeutic option.

A major technical gap limiting both efficacy interpretation and risk stratification is dosimetry. None of the included meta-analyses incorporated individualized (patient-specific) dosimetry. This limitation prevents robust evaluation of dose–response relationships and constrains interpretation of subgroup differences (e.g., resin vs. glass microspheres or tumor-burden strata), because absorbed dose to tumor and non-tumoral liver, key determinants of response and toxicity, cannot be standardized across retrospective series. This is particularly relevant given current EANM guidance emphasizing pre-treatment MAA evaluation and individualized absorbed-dose planning to optimize tumor dose while respecting hepatic tolerance [5]. Future prospective studies should therefore integrate standardized dosimetric reporting as a prerequisite for clinically actionable thresholds and meaningful cross-study comparisons.

From a radiobiological standpoint, SIRT represents a locally delivered radionuclide therapy intended to achieve high absorbed doses within the tumor liver segments while limiting exposure to non-tumoral liver and non-target organs. The dominant clinical effects are expected to be deterministic at the treated sites, whereas toxicity to normal liver and non-target deposition remain key concerns. In practice, this conceptual framework reinforces the clinical relevance of careful angiographic planning, avoidance of non-target embolization, and individualized dose planning to balance tumor control and hepatic tolerance. Although detailed radiation-protection procedures are beyond the scope of this umbrella review, basic principles include minimizing unnecessary exposure to staff and caregivers through standard handling and institutional protocols.

The methodological quality of the included reviews was variable. According to AMSTAR 2 criteria, three were rated as moderate quality and four as low quality. Common methodological shortcomings included a lack of protocol registration, insufficient assessment of risk of bias in primary studies, and failure to evaluate publication bias. Moreover, the vast majority of included studies in the meta-analyses were observational and retrospective, increasing the risk of selection bias and confounding.

Although seven reviews have been included, the structure of the available literature is unbalanced. Many of the more detailed quantitative estimates, particularly the adverse event rates and response stratification, are predominantly determined by the comprehensive meta-analysis by Liu et al. [15]. Consequently, the main added values of this umbrella review lie in a structured assessment of methodological quality (AMSTAR-2: four low-quality and three moderate-quality reviews), the harmonization of clinically relevant heterogeneity, and the explicit identification of gaps that currently prevent dose–response inference and comparative effectiveness.

In summary, while SIRT shows encouraging signals of efficacy and appears safe in selected patients with breast cancer liver metastases, especially those with liver-limited disease and low tumor burden, the current evidence is limited by heterogeneity, methodological weaknesses, and reliance on non-randomized data. There is a clear need for well-designed prospective trials and standardized outcome reporting to better define the role of SIRT in this patient population, in particular, comparing SIRT in addition to systemic therapy with systemic therapy alone.

## 5. Conclusions

SIRT emerges as a potentially valuable treatment for selected patients with breast cancer liver metastases, offering meaningful disease control and acceptable safety in appropriately chosen cases. Outcomes appear most favorable in patients with liver-only or liver-dominant disease and limited hepatic tumor burden, while prognosis is markedly poorer in the presence of extrahepatic metastases or high liver involvement. However, the current evidence base remains largely non-randomized, methodologically variable, and heterogeneous in response criteria and endpoint reporting; consequently, survival outcomes should be interpreted as descriptive rather than evidence of benefit attributable to SIRT. Safety is generally acceptable in pooled analyses, but rare severe events, including RILD-related deaths, have been reported, underscoring the need for careful selection and liver-function evaluation. Future prospective studies should incorporate standardized response assessment, systematic adverse-event collection, and dosimetry-informed planning to clarify its optimal clinical role and integration within multidisciplinary treatment strategies.

## Figures and Tables

**Table 1:** Quality assessment of the included systematic reviews.

N	AMSTAR-2 Criteria	Smits, M.L. et al., 2013 [10]	Feretis, M. et al., 2020 [11]	Rivera, K. et al., 2021 [12]	Guglielmo, P. et al., 2025 [13]	Aarts, B.M. et al., 2021 [14]	Liu, C. et al., 2022 [15]	Quartuccio, N., 2024 [16]
1	Research questions and inclusion criteria include components of PICO	yes	yes	yes	yes	yes	yes	yes
2	Review methods established prior to the conduct of the review (protocol) and deviations justified	no	no	yes	no	yes	no	no
3	Selection of study design explained	partial yes	partial yes	yes	yes	yes	yes	yes
4	Comprehensive literature search strategy	yes	yes	yes	yes	yes	yes	yes
5	Study selection in duplicate	yes	yes	yes	yes	yes	yes	yes
6	Data extraction in duplicate	no	yes	yes	partial yes	no	yes	yes
7	List of excluded studies and justification of the exclusions	partial yes	no	yes	no	yes	yes	yes
8	Included studies described in adequate detail	partial yes	partial yes	partial yes	yes	yes	yes	yes
9	Technique for assessing the risk of bias satisfactory	no	no	no	partial yes	partial yes	yes	no
10	Sources of funding for the primary studies reported	no	no	no	no	yes	partial yes	no
11	Appropriate methods for meta-analysis	partial yes	no	partial yes	partial yes	yes	yes	yes
12	Potential impact of risk of bias results on meta-analysis assessed	no	no	no	no	partial yes	yes	no
13	Risk of bias results accounted for in discussion/conclusion	no	no	no	no	no	yes	yes
14	Satisfactory discussion and explanation of observed heterogeneity, if any	no	no	no	partial yes	yes	yes	yes
15	Adequate investigation of publication bias	no	no	no	no	no	yes	no
16	Conflict of interest of review authors and funding received for conducting the review reported	no	partial yes	yes	yes	yes	yes	yes
	Overall methodological quality	low	low	low	low	moderate	moderate	moderate

**Table 2:** Main characteristics of selected systematic reviews and/or meta-analyses on the efficacy of SIRT in patients with breast cancer.

Authors (Year of Publication)	Studies (Patients) Included	ORR (%)	DCR (%)	Median OS (Months)	Statistical Heterogeneity	Publication Bias
Smits, M.L. et al., 2013 [10]	6 (198)	39–61	78–96	10.8–20.9	NR	NR
Feretis, M. et al., 2020 [11]	12 (452)	NR	81	11.3 (range 3.6–20.9)	NR	NR
Rivera, K. et al., 2021 [12]	9 (380)	NR	NR	11.5 (range 6.6–13.6)	NR	NR
Guglielmo, P. et al., 2025 [13]	8 (298)	44	86	6.1–35.4	NR	NR
Aarts, B.M. et al., 2021 [14]	11 (482)	49 (95% CI 32–67)	NR	9.2 (range 6.1–35.4)	YES (65%)	NR
Liu, C. et al., 2022 [15]	24 (412)	36 (95% CI 26–47)	85 (95% CI 76–93)	9.8 (95% CI: 9.0–11.6)	YES (>75%)	NO
Quartuccio, N. et al., 2024 [16]	18 (650)	51 (95% CI 40–61)	88 (95% CI 82–94)	NR	YES	NR

ORR—objective response rate; DCR—disease control rate; NR—not reported; OS—overall survival; CI—confidence interval.

**Table 3:** Main characteristics of selected systematic reviews and/or meta-analyses on the safety of SIRT in patients with breast cancer.

Authors (Year of Publication)	Administered Activity * (GBq)	Dosimetry	Adverse Events (Grade ≥ 3)	Notes on Safety
Smits, M.L. et al., 2013 [10]	1.64–2.1	NR	Elevation of liver enzymes, transaminase toxicity; nausea and/or vomitus (0–16%); gastric ulcers (0–10%); treatment-related death (0–3%)	No pooled safety data, 3 treatment-related deaths within 3 months post-SIRT, all ascribed to RILD (not reported if histopathologically confirmed)
Feretis, M. et al., 2020 [11]	0.8–2.1	NR	NR	No pooled safety data, TARE considered safe with manageable complications
Rivera, K. et al., 2021 [12]	NR	NR	NR	NR
Guglielmo, P. et al., 2025 [13]	0.8–2.5	NR	NR	No pooled safety data, mostly mild-moderate
Aarts, B.M. et al., 2021 [14]	0.8–3.8	NR	NR	No pooled safety data; reported in 0–54% of patients, not further specified due to heterogeneous definitions across different studies
Liu, C. et al., 2022 [15]	1.6–2.1	NR	Cholecystitis (1.5%); ulcer GI (2.1%); pancreatitis (0.4%); AST/ALT (18.1%); bilirubin (6.3%); ALP (4.4%); leucocytosis (3.3%); anemia/thrombocytopenia (0%)	Pooled safety data
Quartuccio, N., 2024 [16]	1.2–2.4	NR	NR	No pooled safety data

NR—not reported; RILD—radiation-induced liver disease; SIRT—selective internal radiation therapy; GI—gastrointestinal; AST/ALT—aspartate aminotransferase/alanine aminotransferase; ALP—alkaline phosphatase; * data reported inconsistently across the studies.

## Data Availability

The data analyzed in this umbrella review can be found in the cited studies.

## Abbreviations

The following abbreviations are used in this manuscript:

AMSTAR-2	A Measurement Tool to Assess Systematic Reviews 2
BC	Breast cancer
BCLMs	Breast cancer liver metastases
CT	Computed Tomography
SIRT	Selective Internal Radiation Therapy
TARE	Transarterial radioembolization
OS	Overall survival
ORR	Objective response rate
DCR	Disease control rate
